# Different colour predictions of facial preference by Caucasian and Chinese observers

**DOI:** 10.1038/s41598-022-15951-8

**Published:** 2022-07-16

**Authors:** Yan Lu, Kaida Xiao, Jie Yang, Michael Pointer, Changjun Li, Sophie Wuerger

**Affiliations:** 1 Leeds Institute of Textile and Colour, University of Leeds, Leeds, UK; 2 School of Electronics and Information Engineering, University of Science and Technology Liaoning, Anshan, China; 3 School of New Media, Beijing Institute of Graphic Communication, Beijing, China; 4 Department of Psychology, University of Liverpool, Liverpool, UK

**Keywords:** Perception, Colour vision, Psychology and behaviour

## Abstract

Facial colour characteristics convey vital personal information and influence social interactions and mate choices as contributing factors to perceived beauty, health, and age. How various colour characteristics affect facial preference and whether there are cultural differences are not fully understood. Here, we provide a useful and repeatable methodology for skin colour research based on a realistic skin model to investigate the effect of various facial colour characteristics on facial preference and compare the role of colour predictors in Caucasian (CA) and Chinese (CN) samples. Our results show that, although the average skin colour of facial areas plays a limited role, together with colour variation and contrast, there are stronger links between colour and facial preference than previously revealed. We also find large cultural differences in facial colour perceptions; Chinese observers tend to rely more heavily on colour and lightness cues to judge facial preference than Caucasian observers.

## Introduction

Facial preference judgements have a profound impact on diverse important social outcomes, such as mate choices and social decision making, thus it has been studied from various facial perspectives^1,2^. In particular, facial symmetry, averageness and sexual dimorphism have been widely studied over the years from an evolutionary or biological perspective^3,4^. Compared to non-colour related facial traits, the colour appearance of a human face has been relatively less investigated but has gained increasing attention in recent years, which may suggest an important role for facial colour characteristics in any of the preference-related evaluations including facial attractiveness, perceived healthiness, and perceived ageing.

Colour is a perceptual stimulus which is essential in daily life and is often considered in terms of aesthetics^5^. The colour appearance of human faces can change either slowly and continuously due to UV exposure^6^, fruit and vegetable (FV) consumption^7^ or rapidly due to factors such as a change of physical or emotional state, use of coloured cosmetics, or a change in the lighting environment. Skin colour has been suggested to act as a constraint in the evolution of receptoral and postreceptoral visual mechanisms^8^, but see also^9^. As a consequence, skin colour preference has been a subject of great interest in many fields including cosmetology, image capture and reproduction, computer graphics, lighting engineering, etc., where effort has been made to satisfy people’s desire to have a beautiful, healthy-looking or youthful facial appearance^10^.

Different facial colour characteristics have been assessed by previous work, including average facial skin colour^11–16^, local skin colour^17^, skin colour variation^18–20^, and facial colour contrast^21–24^, for their role in facial preference judgements. With a few notable exceptions, these studies generally examined the role of a single colour characteristic in predicting facial preference. The exceptions include a study that compared average skin colour with structural facial features, which showed that skin colour did not predict facial attractiveness^15,25^. Studies that investigated skin colour and various biophysical properties such as wrinkling and sagging on age perception, showed that skin colour had only a weak association with perceived age, while skin colour uniformity was the most important attribute^26,27^. Tan et al. used cropped cheek skin images to investigate the role of both skin colour and skin colour variation on health perception among Malaysian Chinese and claimed that homogenous skin texture and increased skin yellowness positively predicted the rated health^28^. The results are mixed, and none considered all the different colour characteristics at the same time. It is not known how these colour characteristics taken together affect facial preference, whether they are correlated themselves, and which characteristics are more important in terms of predicting facial preferences including attractiveness, healthiness, and visual age. One aim of the present study is to investigate the effect of various colour characteristics on facial preference evaluation and identify their relative contributions in predicting facial preference.

More importantly, the existing studies on the same colour predictors generated controversial results, potentially due to the different methodologies. Studies in which facial skin colour was experimentally manipulated reported generally much stronger associations between facial colour characteristics and preference have been revealed compared to recent studies using non-manipulated facial images^15,16,28–30^. In the former studies, observers were asked either to manipulate the facial colour to enhance their perceived preference or to rate or make a preference choice between the colour-manipulated facial images. These studies concluded that increased facial skin lightness, redness and yellowness significantly enhance healthy appearance and facial attractiveness, mostly for Caucasian people^11–14,31,32^. A small number of more recent studies employed non-manipulated real facial images for preference evaluation and revealed very weak correlations between average skin colour and perceived healthiness (p > 0.636)^15^, a limited role for colour in predicting attractiveness (p > 0.05)^25^, and much weaker associations between skin colour and perceived age compared to skin colour uniformity or distribution^26,27^.

Although image manipulation could be an effective way to explore the effect of one single variable on preference evaluation while holding all other variables constant, it may not be a useful method to exam the interplay between various variables and the role of an isolated colour characteristic may be overestimated. Facial skin colour manipulations sometimes include uniform colour shifts applied across the entire face, or may result in colour manipulations outside the natural skin colour gamut. Moreover, the computer-generated or morphed facial images may lose skin texture and appear to be unrealistic after image processing.

**Figure 1:**
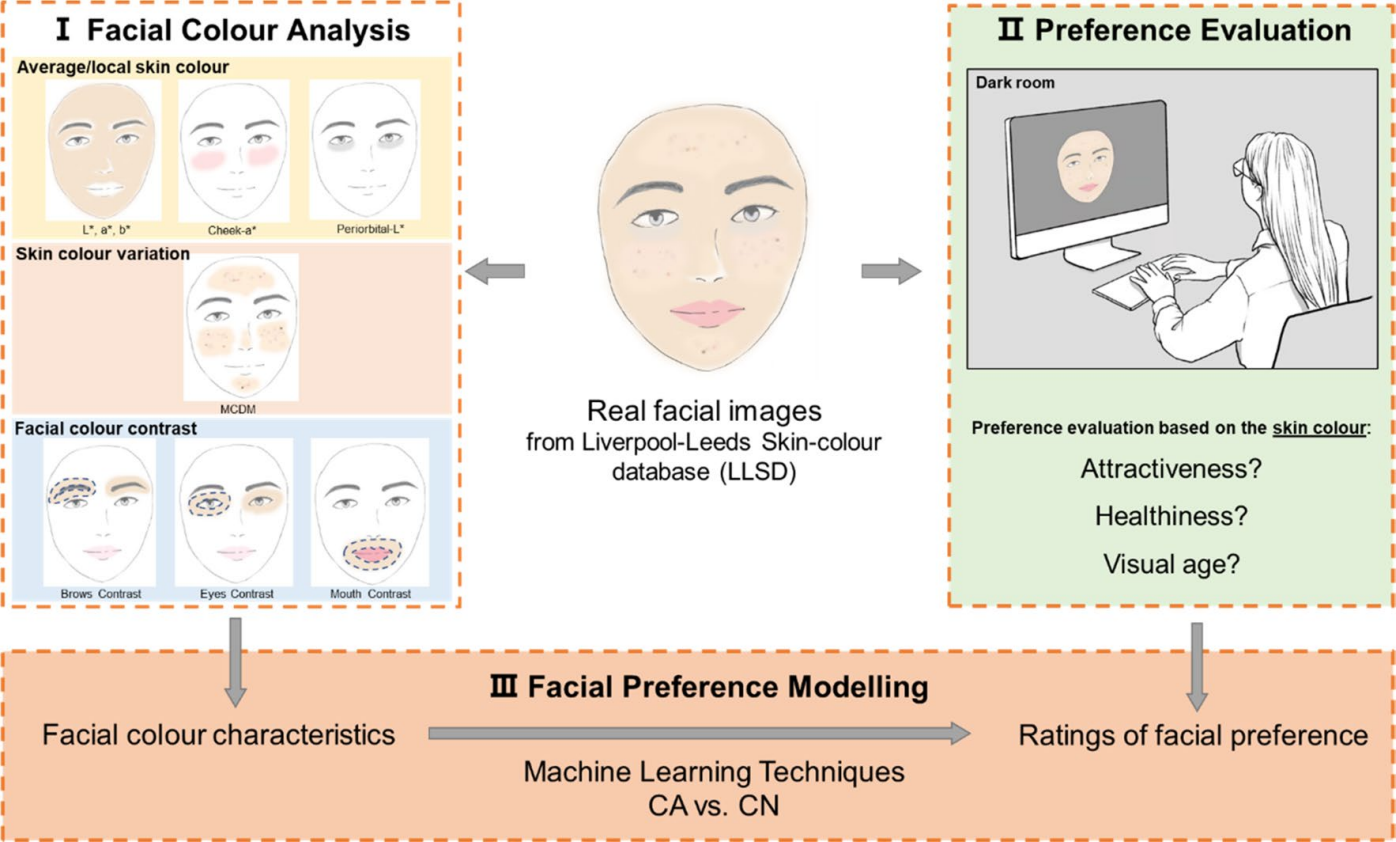
A schematic diagram of the key idea in this study. I. Analysis of various facial colour characteristics of 80 real facial images from LLSD II. Observers evaluate the colour appearance of the real facial images in terms of the three attributes of facial preference: attractiveness, perceived healthiness, and visual age. III. Machine learning techniques are used in modelling to predict facial preference from colour predictors. The cultural difference is investigated between Caucasian and Chinese populations.

Considering all the above, the present study aims to study facial colour preference within an evolutionary meaningful parameter space, and to provide a useful and repeatable methodology for skin color research based on a realistic skin model. Figure 1 shows the schematic diagram of the methodology used in this study. We use a set of high-resolution images of real human faces without changing the original colour. Colour analyses are performed on each of the facial images and a robust process of colour characterization for both the camera and the display is performed to truly present facial colour appearance in the preference evaluation experiments.

While the effect of facial colour characteristics on preference judgement has been most studied using Caucasian examples, both as participants and to provide stimulus material, there are a few cross-cultural studies. Stephen et al. and Coetzee et al. conducted studies amongst Caucasian and African populations and demonstrated similar preferences for skin colour in relation to perceived health and attractiveness^32–34^. A study conducted by Han et al., however, did not find a cross-cultural similarity in facial colour preference but reported different preferences between Mainland Chinese and Caucasians: Chinese observers prefer lighter skin and decreased yellowness compared to Caucasian participants^35^. Malaysian Chinese observers, by contrast, associated increased yellowness and redness, but decreased lightness with enhanced perceived healthiness^36^. Our previous study also concluded that skin colouration is not a universal but culturally-specific cue for attractiveness, healthiness, and youthfulness in observers of Chinese and Caucasian ethnic groups^16^. Note that only the average skin colour was considered in the studies described above, whereas in the present study, cultural differences between Chinese and Caucasian samples are further explored taking into account a range of facial colour characteristics.

The objectives of the present study are: (1) to evaluate the role of different facial colour characteristics in predicting preference using non-manipulated images of real faces; (2) to identify the most important colour characteristics for each of the three facial preference attributes: attractiveness, healthiness, and visual age; (3) to investigate the cultural difference on preference judgement between Chinese and Caucasian observers. To achieve these objectives, colour characteristics including average/local skin colour, skin colour variation, and facial colour contrast are measured using non-manipulated images of both real Caucasian and real Chinese faces. A rating study is conducted, using both Caucasian and Chinese observers, to obtain preference evaluations including facial attractiveness, perceived healthiness, and visual age. Separate data analyses are carried out for each ethnic group to examine the role of colour in predicting the preference rating of their own faces. We rely on the techniques from machine learning and provide a comprehensive assessment of the relative importance of various facial colour characteristics that contribute to facial attractiveness, perceived healthiness, and perceived age.

Our results reveal a moderate role for colour characteristics in determining facial preference. Although the average facial skin colour plays a limited role, together with colour variation and contrast, there are stronger links between colour and facial preference than previously revealed. Moreover, different facial colour cues are found to be utilized by different observers according to the different preference attributes they are accessing. Interestingly, Chinese observers tend to rely more heavily on colour cues to judge all facial preference attributes than Caucasian observers. The results highlight the importance of examining various facial colour cues simultaneously to characterise the role of colour predictors for in facial preference evaluation and demonstrate the large cultural difference between Caucasian and Chinese populations.

## Results

### Variation in facial colour characteristics across Caucasian (CA) and Chinese (CN) images

**Figure 2:**
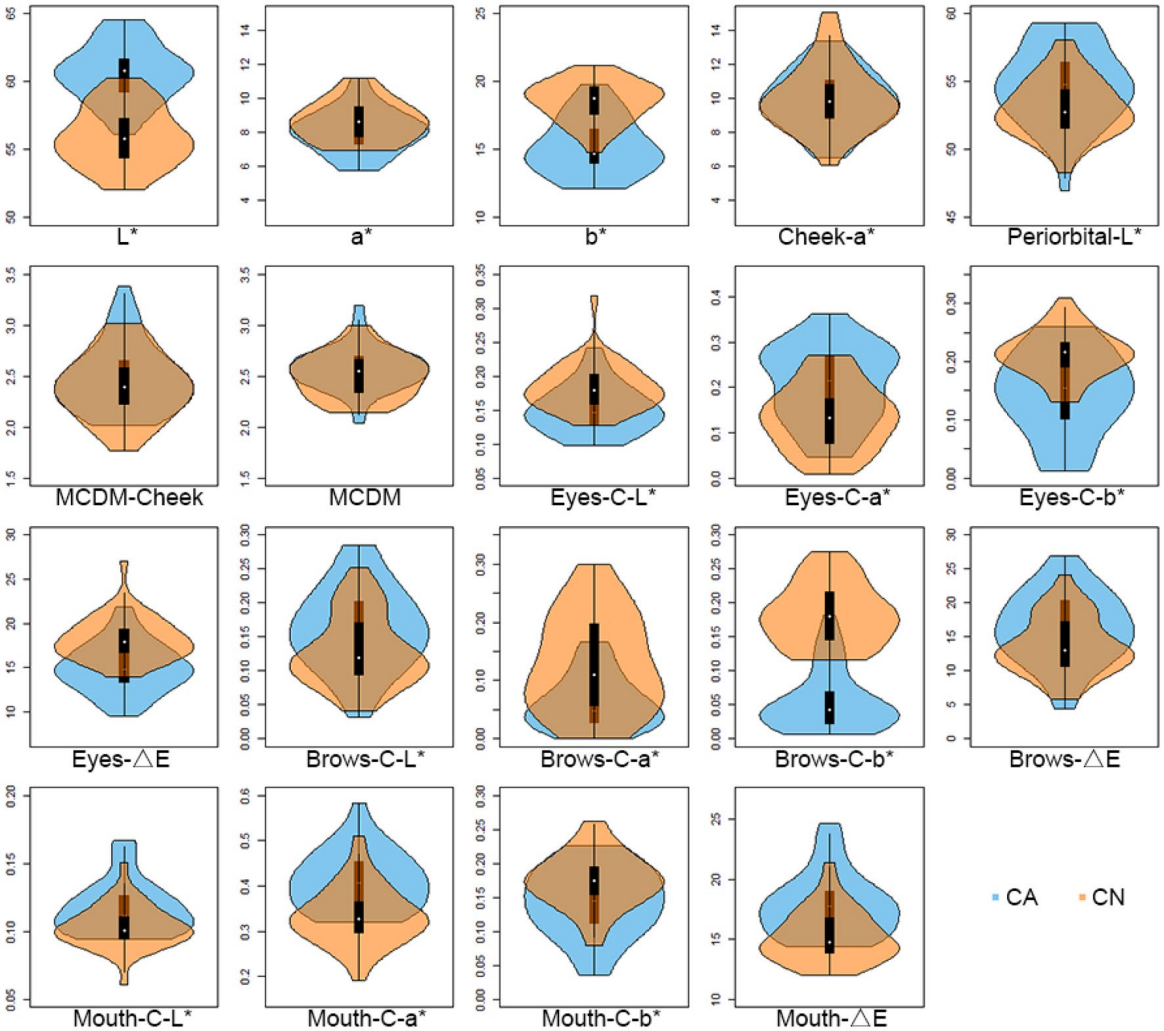
Violin plots showing range and variation of facial colour characteristics in CA and CN facial images. White points indicate medians, black rectangles represent interquartile ranges.

All the facial colour characteristics are quantified in CIELAB colour space, which is designed to be perceptually uniform. Figure 2 shows all the parameters measured for the forty CA faces and the forty CN faces. The lightness and colour variations in these images are representative of the colour variations in the respective populations^37^. CA and CN faces differ in various facial colour characteristics. The mean values and standard deviations for each group can be found in Appendix 1, together with the results of a two-sample *t*-test (P values) for the difference between the two ethnic datasets. All colorimetric characteristics between the two ethnic sample differ statistically from each other (P < 0.05), except for the cheek redness (cheek-a*) and the skin colour variations (MCDM-cheek and MCDM). The mean scores and standard deviations of all three preference ratings for both datasets are also given in Appendix 1.

### Zero-order correlations between facial colour characteristics and each facial preference

**Figure 3:**
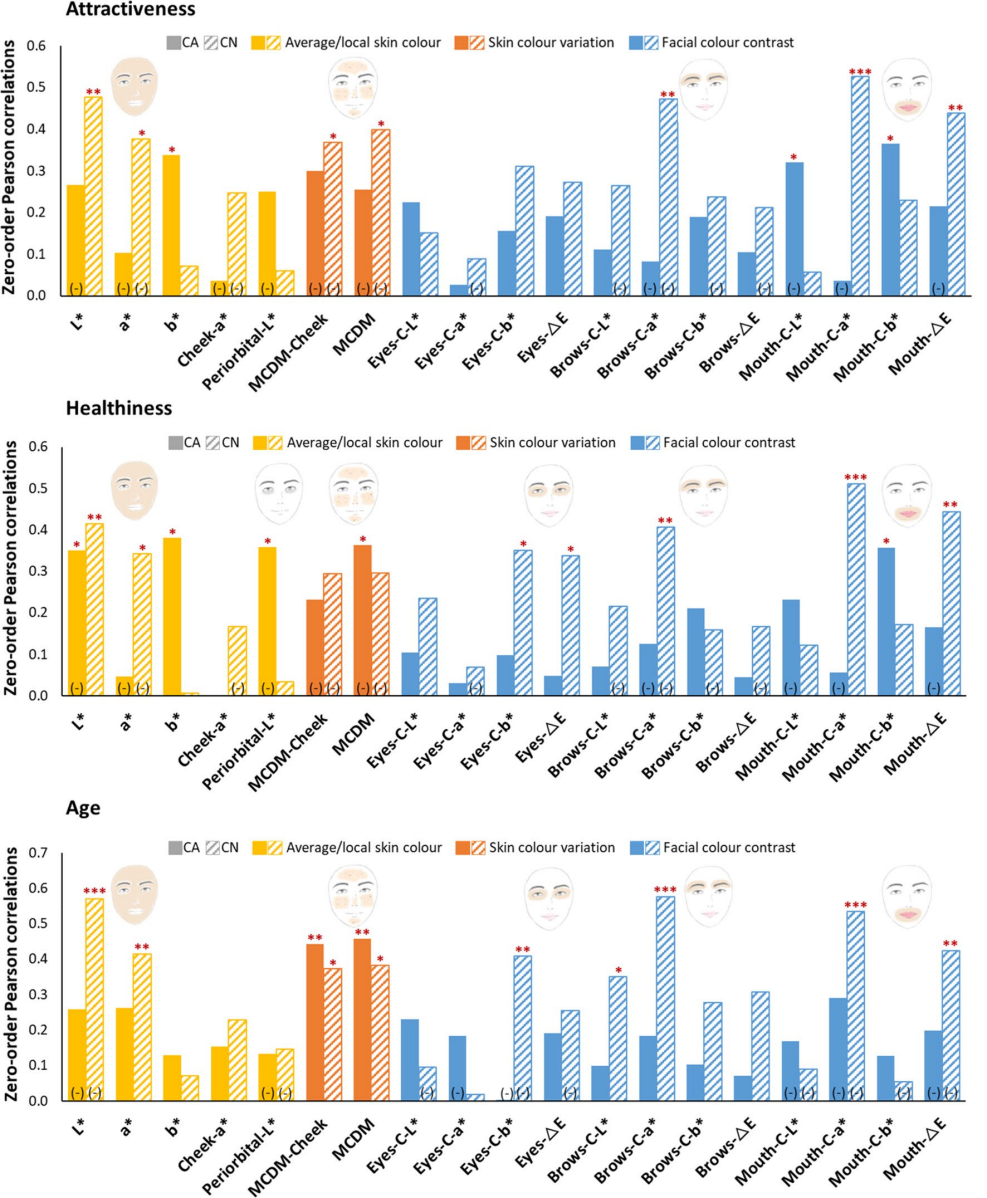
The Pearson Correlations between each facial colour characteristic and each facial preference attributes: attractiveness (top), healthiness (middle), and age (bottom). Each bar chart represents the correlation coefficient (left darker bar chart: CA; right lighter bar chart: CN); all the negative coefficients are marked with (–) at the bottom of the bar charts; Asterisks above the bar charts indicate the statistical significance of each relationship: *p ≤ 0.05, ** p ≤ 0.01, ***p ≤ 0.001.

The Pearson Correlation Coefficient (two-tailed) was used to identify correlations between each colour characteristic and facial attractiveness, healthiness, and visual age rated by the observers, for the Caucasian and Chinese datasets, respectively. The results for each of the three preference ratings are shown in Fig. 3 and the complete correlation matrix of preference ratings and facial colour characteristics can be found in Appendix 2.

#### Facial attractiveness

As shown in Fig. 3, facial colour characteristics are linked differently with facial attractiveness by the Caucasian (solid bars) and the Chinese observers (dashed bars). In the Caucasian dataset, facial attractiveness was positively correlated with facial yellowness (b*, p < 0.05) and b* contrast around the mouth (mouth-C-b* p < 0.05), but negatively with L* contrast around the mouth (mouth-C-L*). In the Chinese dataset, facial attractiveness was positively correlated with facial lightness (L*, p < 0.01), a* contrast around the mouth (mouth-C-a*, p < 0.001), and colour difference around the mouth (mouth-∆E, p < 0.01), which may also result from the a* contrast considering the high correlation between a* contrast and △E around the mouth (r = 0.859, p < 0.001 in ESM Appendix 2). Chinese facial attractiveness is negatively correlated with facial redness (a*), both skin colour variation (MCDM-cheek and MCDM), and a* contrast around the brows (brows-C-a*).

#### Perceived healthiness

The attractiveness ratings and healthiness ratings are highly correlated for both groups (r > 0.9, p < 0.001 in Appendix 2), thus colour cues utilized for healthiness perception are somewhat similar to those for attractiveness judgements. For the Caucasian dataset, perceived healthiness is positively correlated to facial yellowness (b*, p < 0.05) and b* contrast around the mouth (mouth-C-b*, p < 0.05), but negatively correlated to overall lightness (L*, p < 0.01), periorbital lightness (periorbital-L*, p < 0.05), and overall skin colour variation (MCDM, p < 0.05). For the Chinese dataset, perceived healthiness is positively correlated to facial skin lightness (L*, p < 0.01), colour contrast around the eye and the mouth (eyes-C-b*, ∆E, p < 0.05; mouth-C-a*, ∆E, p < 0.01). Perceived healthiness for the Chinese dataset is negatively correlated with facial redness (a*, p < 0.05) and a* contrast around the brows (brows-C-a*, p < 0.01).

#### Perceived age

For the Caucasian dataset, perceived age is only significantly and positively associated with skin colour variation (MCDM-cheek and MCDM, both p < 0.01), which means larger variation in Caucasian skin colour is linked to older visual age. For the Chinese dataset, in addition to skin colour variation (MCDM-cheek and MCDM, both p < 0.05), perceived age is also positively correlated with facial redness (a*, p < 0.01), colour contrast around the brows (brows-C-L*, a*, p < 0.05). In addition, it is negatively correlated with facial lightness (L*, p < 0.001), colour contrast around the eye and mouth (eyes-C-b*, p < 0.01; mouth-C-a*, p < 0.001; mouth-∆E, p < 0.01).

### The separate model: comparisons of three classes of facial colour characteristics in determining facial preference

**Figure 4:**
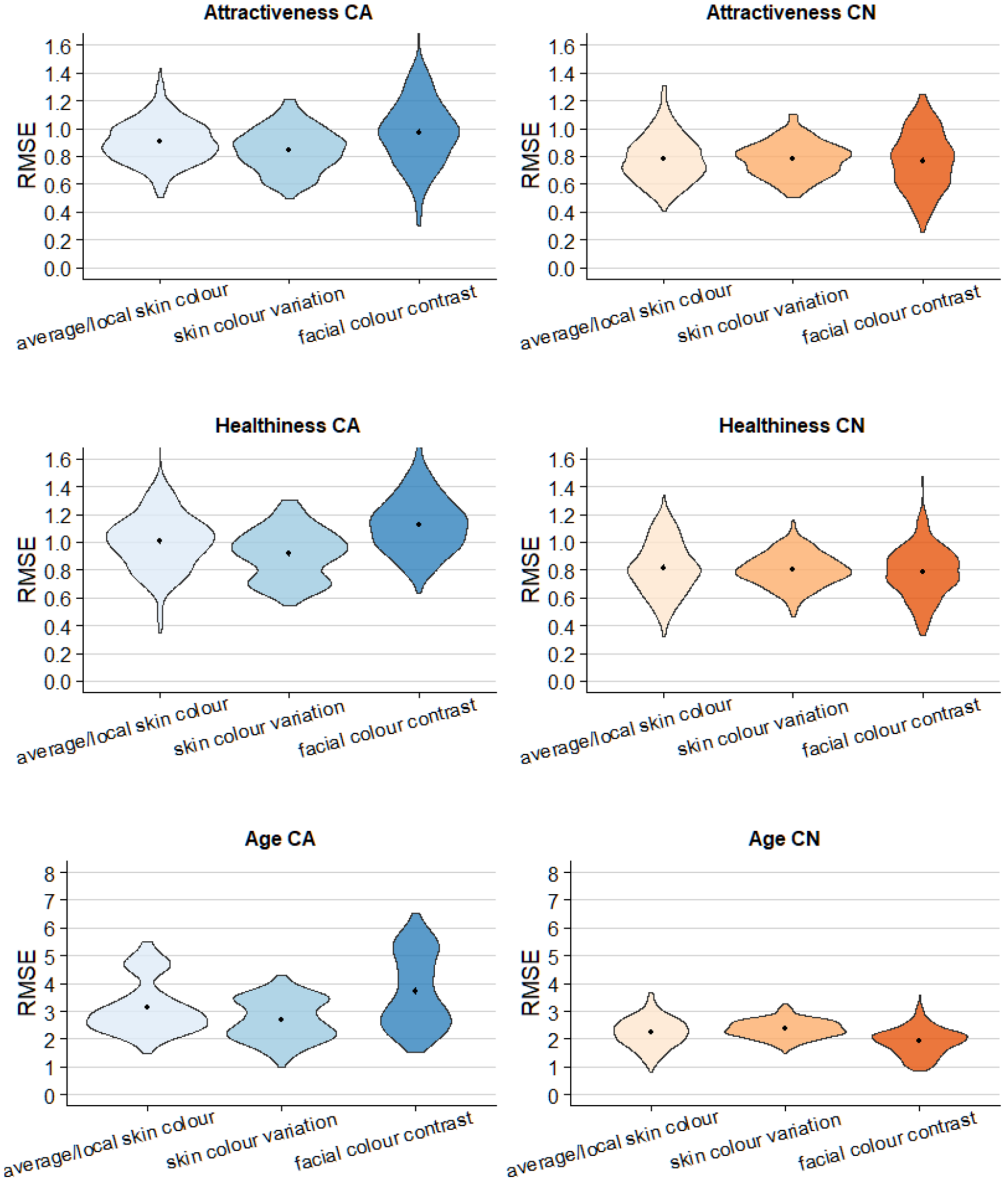
The model performance of the three classes of facial colour characteristics in predicting each facial preference attributes: attractiveness (top), healthiness (middle), and age (bottom). CA results are in the left column and CN results are in the right column. Black dots indicate the mean RMSE from fivefold cross-validation with 50 repeats.

To further investigate the role of the three different classes of colour characteristics (average/local skin colour, skin colour variation, and facial colour contrast) in predicting the preference of real human faces, and identify their relative importance, techniques from machine learning were implemented, following previous studies^38,39^. We used cross-validated linear regression models (fivefold cross validation with 50 repeats) to compare the predictive power of the three different classes of facial colour characteristics. Each model’s overall predictive fit was assessed by the mean RMSE (root mean square error) over all splits, as shown in Fig. 4.

#### Facial attractiveness

For the Caucasian dataset, the model of skin colour variation showed the best predictive accuracy (M_RMSE_ = 0.85, SD_RMSE_ = 0.15), followed by the model of average/local skin colour (M_RMSE_ = 0.90, SD_RMSE_ = 0.15), and facial colour contrast (M_RMSE_ = 0.97, SD_RMSE_ = 0.24). For the Chinese dataset, the three classes showed similar predictive accuracy (average/local skin colour: M_RMSE_ = 0.78, SD_RMSE_ = 0.17; skin colour variation: M_RMSE_ = 0.78, SD_RMSE_ = 0.12; M_R_^2^ = 0.21 facial colour contrast: M_RMSE_ = 0.77, SD_RMSE_ = 0.21).

#### Perceived healthiness

For the Caucasian dataset, the model of skin colour variation showed the best predictive accuracy (M_RMSE_ = 0.91, SD_RMSE_ = 0.18), followed by the model of average/local skin colour (M_RMSE_ = 1.01, SD_RMSE_ = 0.18), and facial colour contrast (M_RMSE_ = 1.10, SD_RMSE_ = 0.20). For the Chinese dataset, the facial colour contrast showed the best predictive accuracy (M_RMSE_ = 0.78, SD_RMSE_ = 0.18), followed by the skin colour variation (M_RMSE_ = 0.80, SD_RMSE_ = 0.12), and the average/local skin colour (M_RMSE_ = 0.81, SD_RMSE_ = 0.19).

#### Perceived age

For the Caucasian dataset, the model of skin colour variation showed the best predictive accuracy (M_RMSE_ = 2.69, SD_RMSE_ = 0.73), followed by the model of average/local skin colour (M_RMSE_ = 3.19, SD_RMSE_ = 0.93), and facial colour contrast (M_RMSE_ = 3.82, SD_RMSE_ = 1.31). For the Chinese dataset, the model of facial colour contrast showed the best predictive accuracy(M_RMSE_ = 1.92, SD_RMSE_ = 0.49), followed by the model of average/local skin colour (M_RMSE_ = 2.22, SD_RMSE_ = 0.53), and the skin colour variation (2.35, SD_RMSE_ = 0.32).

### The combined model: role of facial colour characteristics in determining facial preference

Finally, we used elastic net regression^40,41^ to evaluate the role of all sixteen facial colour characteristics in determining facial preference by simultaneously entering them into one regression model. The colour difference, ∆E, around the three facial features (eyes, brows, mouth-∆E) was excluded since it has originated from one of the separate colour contrast channels (L*, a*, or b*) for both groups according to the correlations in ESM Appendix 2 (r > 0.86, p < 0.001). We also implemented cross validation to first generate the optimal combination of the two model hyperparameters, α and λ, with maximized fit (minimized RMSE) and then test the model fit with the optimal α and λ by the mean RMSE over all splits. The performance of each combined model represented by the mean RMSE and the relative importance of different facial colour characteristics in the model represented by the absolute β values were reported below.

#### Facial attractiveness

**Figure 5:**
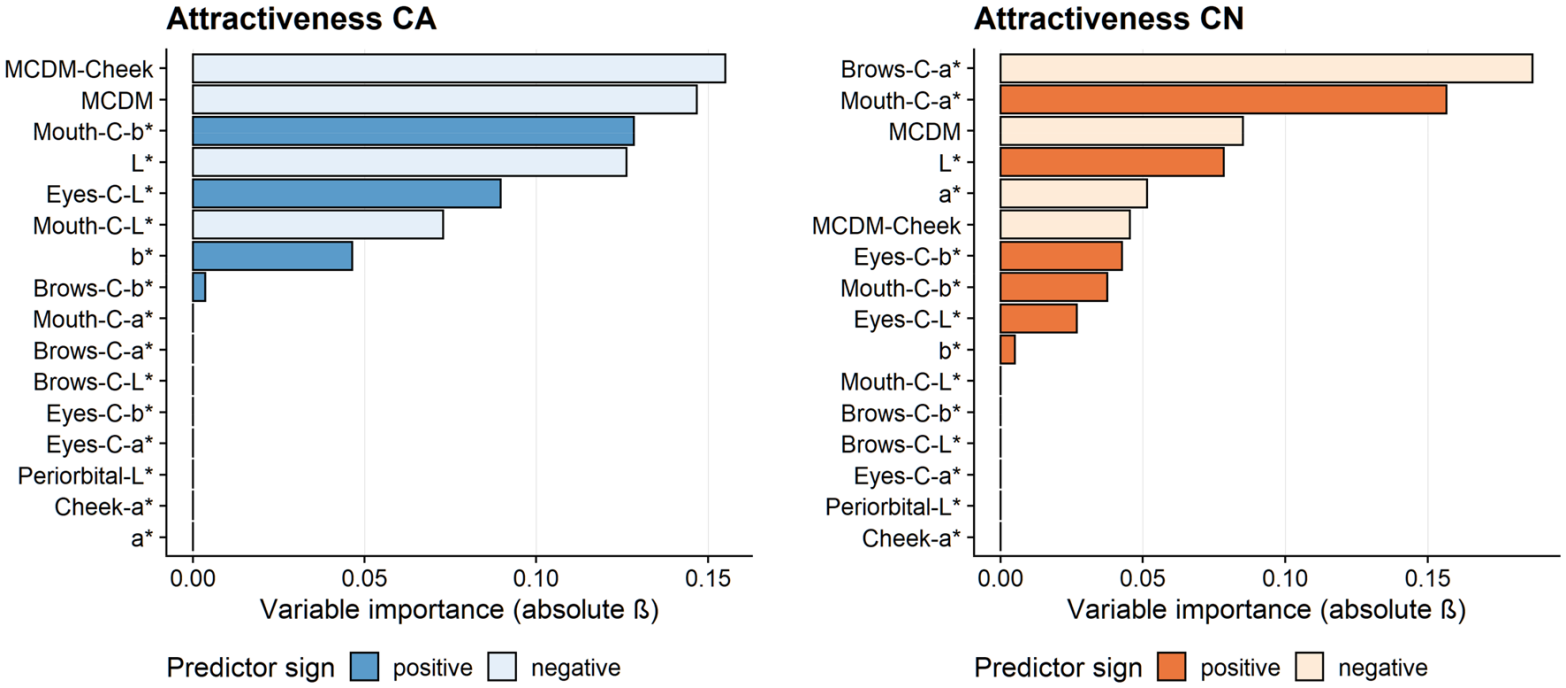
The relationship between different facial colour characteristics and facial attractiveness. CA results are in the left and CN results are in the right. Coefficients were derived from the elastic net model with fivefold cross validation and 50 repeats.

For the Caucasian dataset, the combined model predicted facial attractiveness within 0.84 point on a 7-point scale (M_RMSE_ = 0.84, SD_RMSE_ = 0.16). As shown in Fig. 5, the skin colour variation (MCDM-cheek, $\overline{\beta }$ = − 0.155 and MCDM, $\overline{\beta }$ = − 0.147) were the strongest predictors, with less skin colour variation predicting higher facial attractiveness. The mouth colour contrast (mouth-C-b*, $\overline{\beta }$ = 0.128) and the facial lightness (L*, $\overline{\beta }$ = − 0.126) were also relatively informative predictors, whereas the brows contrast ($\overline{\beta }$ = 0.003) was relatively uninformative. For the Chinese dataset, the combined model predicted facial attractiveness within 0.71 point on a 7-point scale (M_RMSE_ = 0.71, SD_RMSE_ = 0.14). The brows colour contrast (brows-C-a*, $\overline{\beta }$ = − 0.187) and the mouth colour contrast (mouth-C-a*, $\overline{\beta }$ = 0.157) were the strongest predictors of facial attractiveness. The facial skin yellowness (b*, $\overline{\beta }$ = 0.005) was relatively less informative.

#### Perceived healthiness

**Figure 6:**
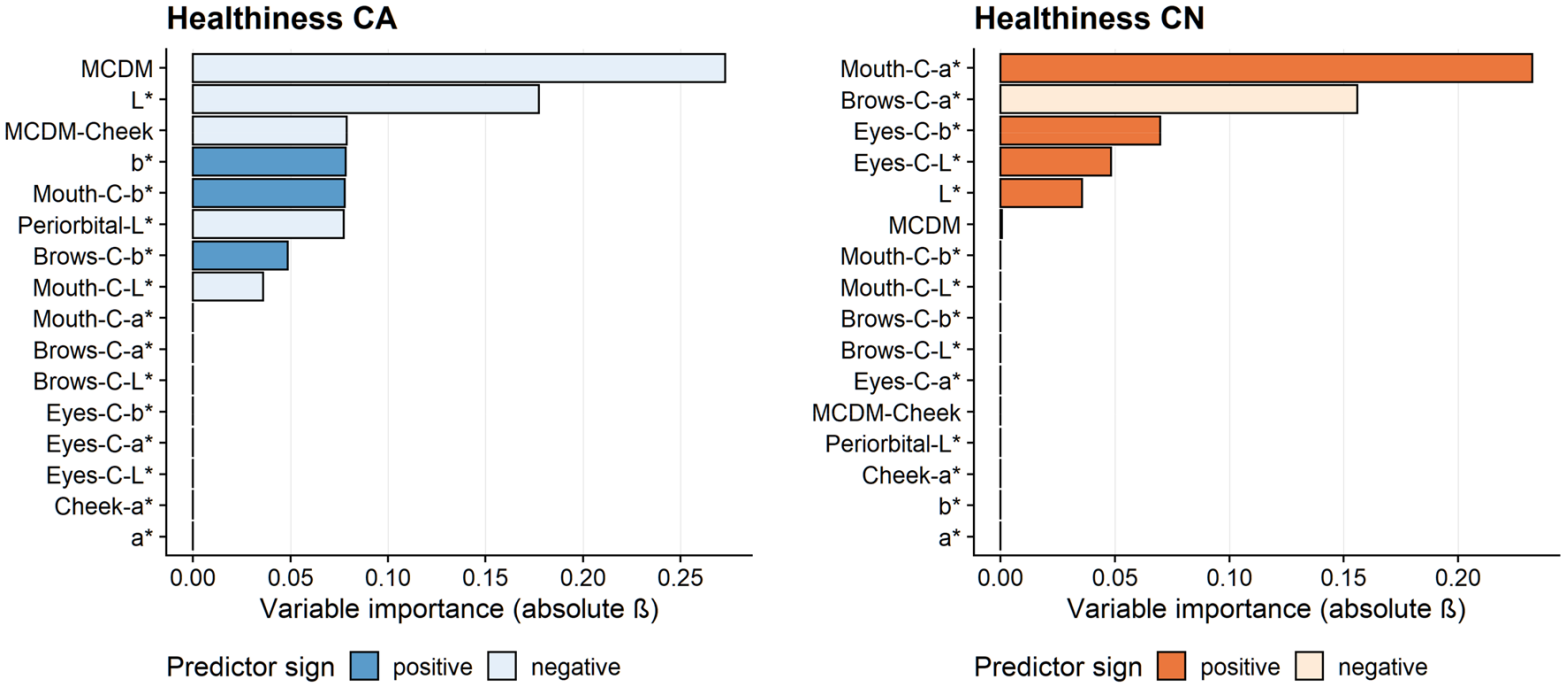
The relationship between different facial colour characteristics perceived healthiness. CA results are in the left and CN results are in the right. Coefficients were derived from the elastic net model with fivefold cross validation and 50 repeats.

For the Caucasian dataset, the combined model predicted perceived healthiness within 0.85 point on a 7-point scale (M_RMSE_ = 0.85, SD_RMSE_ = 0.16). As shown in Fig. 6, the overall skin colour variation (MCDM, $\overline{\beta }$ = − 0.273) and the facial lightness (L*, $\overline{\beta }$ = − 0.177) were the strongest predictors, with less skin colour variation and lower skin lightness predicting higher perceived healthiness. The mouth luminance contrast (mouth-C-L*, $\overline{\beta }$ = − 0.036) was relatively uninformative. For the Chinese dataset, the combined model predicted facial attractiveness within 0.73 point on a 7-point scale (M_RMSE_ = 0.73, SD_RMSE_ = 0.11). The mouth colour contrast (mouth-C-a*, $\overline{\beta }$ = 0.232) and the brows colour contrast (brows-C-a*, $\overline{\beta }$ = − 0.156) were the most strongest predictors of perceived healthiness. The overall skin colour variation (MCDM, $\overline{\beta }$ = − 0.001) was relatively less informative.

#### Perceived age

**Figure 7:**
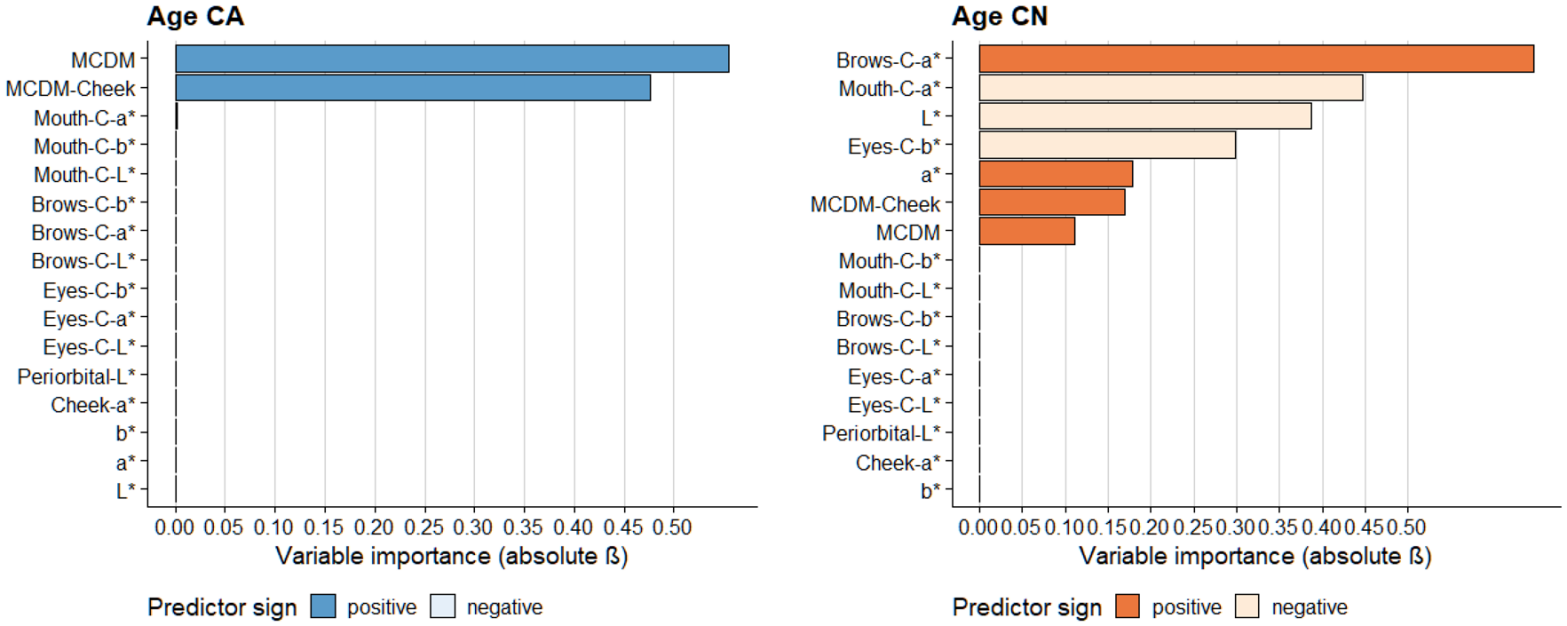
The relationship between different facial colour characteristics and perceived age. CA results are in the left and CN results are in the right. Coefficients were derived from the elastic net model with fivefold cross validation and 50 repeats.

For the Caucasian dataset, the combined model predicted perceived age within 2.88 years on a single-year step scale from 1 to 99 years (M_RMSE_ = 2.88, SD_RMSE_ = 0.99). As shown in Fig. 7, the skin colour variation (MCDM, $\overline{\beta }$ = 0.555 and MCDM-cheek, $\overline{\beta }$ = 0.476) were the strongest predictors for perceived age, with a larger skin colour variation predicting a higher estimated age. The mouth colour contrast (mouth-C-a*, $\overline{\beta }$ = − 0.002) was relatively uninformative. For the Chinese dataset, the combined model predicted perceived age within 1.83 years on a single-year step scale from 1 to 99 years (M_RMSE_ = 1.83, SD_RMSE_ = 0.34). Similar to the perceived healthiness, the brows colour contrast (brows-C-a*, $\overline{\beta }$ = 0.646) and the mouth colour contrast (mouth-C-a*, $\overline{\beta }$ = − 0.446) were also the most strongest predictors of perceived age. The facial lightness (L*, $\overline{\beta }$ = − 0.387) and the eyes colour contrast (eyes-C-b*, $\overline{\beta }$ = − 0.298) were also relatively informative predictors, whereas the overall skin colour variation (MCDM, $\overline{\beta }$ = 0.110) was relatively less informative.

## Discussion

The present study provides a useful and repeatable methodology for the comprehensive assessment of various facial colour characteristics that affect facial preference. Colour predictors of facial attractiveness, perceived healthiness, and perceived age were studied in both Caucasian and Chinese samples. Our findings show that the three classes of facial colour characteristics (average skin colour, skin colour variation, facial colour contrast) are of similar importance in facial preference judgements. It has also addressed the cultural difference between Caucasian and Chinese observers in that Chinese observers tend to rely more heavily on colour cues to judge facial preference than Caucasian observers.

### Colour predictors for facial attractiveness and perceived healthiness

For both Caucasian and Chinese observers, a part of colour predictors of attractiveness and perceived healthiness are overlapping since these two perceptual ratings were highly correlated for both datasets (r = 0.912 for CA dataset, r = 0.927 for CN dataset, see Appendix 2).

For Caucasian observers, skin colour variation is the strongest predictor to rate attractiveness and perceived healthiness, and more evenly distributed skin colour with less variation is linked to enhanced facial attractiveness and perceived healthiness, which is consistent with previous studies^19,20^. Compared to the skin colour variation, the averaged skin colour is less important and only the L*, b* were found to be predictors of attractiveness and healthiness. Decreased facial lightness and increased facial yellowness enhance Caucasians’ facial attractiveness and perceived healthiness, which could be explained by the melanin- and carotenoid-linked health-signalling system^11,33^. In contrast to previous reports, facial redness (a* or cheek-a*) is not an important predictor for Caucasian preference,- corroborating our previous work^16^ and may be due to the small range of naturally occurring skin colour variation and thus the observers focus more on the other colour cues when rating the real facial images. In the current study, facial colour contrast did not emerge as an important predictor of preference in Caucasians, in contrast to previous studies^21,22,24^; only contrast around the mouth showed a limited role in attractiveness and perceived health. The reason for this is also likely to be the limited range of facial colour contrast in real faces without any applied cosmetics.

For Chinese observers, facial colour (a*) contrast (brows-C-a*, mouth-C-a*) is the most important predictor among different colour characteristics to judge both attractiveness and healthiness. Facial lightness (L*) is another consistent cue for Chinese people to judge facial preference. In contrast to Caucasians’ preference for decreased skin lightness, Chinese observers associate increased facial lightness with enhanced facial attractiveness and healthiness, in line with previous studies^35^. The opposite preference for skin tanning^11^ suggests the mainstream aesthetic difference between the two cultures. Skin colour variation also emerged as a predictor for Chinese observers but only when they judge facial attractiveness, with smaller variation in skin colour linked to enhanced facial attractiveness. Local skin colour does not emerge as a relevant predictor when all colour features are considered together.

### Colour predictors for perceived age

Skin colour variation is found to be a predictor of perceived age in both the Caucasian and Chinese datasets (Fig. 7). Crucially, it is the only important colour cue for age perception of Caucasian observers judging own-ethnicity faces. Larger variation in facial/cheek skin colour is linked to older visual age. This is in agreement with the study of Nkengne et al., which looked at the influence of various skin attributes (skin yellowness, skin texture, etc.) on the age perception of Caucasians and found that skin colour uniformity was the most important attribute. Chinese observers deploy the colour cues differently from the Caucasian sample. Since all three perceptual ratings from Chinese observers are highly correlated (r > 0.818, in ESM Appendix 2), the significant colour predictors of perceived age are similar to the predictors of attractiveness and healthiness. For Chinese observers, facial redness contrasts (brows and mouth) are the most important predictors for perceived age, but are deployed differently: brow and mouth contrasts are associated with a decrease and increase in youthfulness respectively. Skin lightness (L*) is the third informative cue for perceived age: a higher facial lightness is associated with youthfulness (younger visual age). Similarly to Caucasian observers, Chinese observers also rate more evenly distributed skin colour as younger. However, skin colour variation only plays a limited role compared to skin colour and contrast.

These results reveal the importance of facial colour (a*) contrast for the Chinese observers. Using the same set of facial colour contrasts, Porcheron et al. investigated their relationship with the perceived age in Chinese subjects and found the mouth a* contrast also had significant and negative correlation with real age and the brows a* contrast had positive correlations with age^42^.

### Cultural difference between Caucasian and Chinese observers

As noted above, the use of different facial colour cues is ethnicity specific (Figs. 5, 6, 7) and our current study extends our previous report on the ethnicity specific use of the average facial skin colour ^16^. Moreover, the cultural differences include the opposite preference for facial lightness and the different importance of the three classes of colour traits (average skin colour, skin colour variation, and facial colour contrast) in preference evaluation. The difference reflects the aesthetic difference between western and eastern culture, which might result from the development of multiple social and cultural factors over a long period of time. Meanwhile, the differential use of the facial colour cues could also stem from the different colorimetric parameters of the faces of the two ethnic groups (as shown in Fig. 2).

Generally, Chinese observers tend to utilize facial colour cues more effectively when evaluating facial preference (attractiveness, perceived healthiness, and visual age) compared to Caucasians, which is reflected in the higher number of significant correlations between perceptual ratings and colour characteristics in the Chinese dataset (Fig. 3). The results of the separate models suggest that all the three classes of colour predictors show better predictive accuracy (smaller RMSE) in the Chinese models compared to the Caucasian models no matter which preference attribute is judged (Fig. 4). Most importantly, the Chinese combined models also give better predictive accuracy than the Caucasian combined models in all preference attributes, which predict attractiveness, healthiness, and visual age within 0.71 point, 0.73 point, and 1.83 years, respectively (the predictive accuracy is 0.84 point, 0.85 point, and 2.88 years for Caucasian model, respectively). These results suggest an important and novel aspect of the cultural difference between Caucasian and Chinese samples. Coetzee et al. investigated the role of facial shape cues and colour cues on attractiveness preference of White Scottish and Black South African people and found that Black South Africans rely heavily on colour cues while White Scottish use shape cues^34^. Given that Asians were less influenced by some structural facial features than Caucasians^43^, we speculate that Caucasians may make facial preference judgements based on more structural facial features than colour cues while Chinese rely more heavily on facial colour cues.

### The role of facial colour characteristics on preference evaluation in real faces

In the present study, 80 calibrated non-manipulated images of real Caucasian and Chinese human faces are used, with colour characteristics that are representative of the naturally occurring variations in these ethnicities. Our study shows the similar importance of all three classes of colour traits (average skin colour, skin colour variation, facial colour contrast) in determining facial preference judgements (Fig. 4). Which colour characteristics are used depends on the preference attribute under consideration and also on the ethnic group (Figs. 5, 6, 7).

Earlier studies using manipulated images have commonly found more significant relationships between the single manipulated colour cue and preference ratings^17,31,42,44,45^. As outlined in the introduction, methodological differences may play a role in estimating the role of certain colour characteristics for preference judgments. When judging facial preference of real human faces, it may be beneficial to consider a wide range of facial colour cues simultaneously, hence allowing an estimate of the relative importance of the individual cues. Considering the importance of facial colour preference in various applications, we attempted to provide a robust method to assess the role of a wide range of facial colour characteristics on real human faces within an evolutionary meaningful parameter space.

More recent studies have started to use non-manipulated images to study facial preference, and found much weaker associations between skin colour and facial preference^15,16,25,26,28,46^. Our previous study found that both Chinese and Caucasian observers make use of average skin colour and lightness to rate attractiveness, healthiness, and perceived age, but to a lesser degree than previously thought^16^. Foo et al. investigated skin colour (L*, a* and b*) and other structural facial features as the preference predictors, and they concluded that skin colour did not predict attractiveness while facial yellowness played a limited role in predicting healthiness^25^. Jones et al. also compared facial shape cues and colour cues in health perception using average facial L*, a*, and b*, and they found no role of skin colour as a short-term health cue ^15^. Tan et al. studied skin texture and colour in health perception and found homogenous skin texture and increased skin yellowness was positively associated with perceived health of Malaysian Chinese faces, however, facial colour contrast was not considered in their study which may also be an important predictor^28^. Consistent with those studies that used non-manipulated images, our results show that average skin colour (L*, a*, and b*) itself, as a single factor, is not a very strong predictor for facial preference evaluation but plays a limited role, especially for Caucasians’ age perception. Given that different facial colour cues were utilized differently depending on the preference judgement at hand and the observers, a wide range of facial colour characteristics need to be studied at the same time to obtain a realistic estimate of the role of colour features for aesthetic preferences.

### Limitations of this study

Our work has several limitations which need to be addressed by future research. Although all the Chinese observers were from and lived most of their lives in mainland China, they all had the short-term experience of study or work in the UK. It is not clear if such experience would affect their aesthetic preference. Future research could address the question by repeating the experiments in mainland China and using native Chinese observers. Furthermore, while the observers were instructed to make judgements based on facial skin colour only, the influence of other cues such as facial shape features cannot be excluded. In our study, we tried to tackle this issue by using a relatively large (80) set of images of real faces, 40 Caucasian and 40 Chinese. Future studies would benefit form including an even larger number of facial images in order to cover the variation of facial shape characteristics and disentangle the role of colour and shape features on preference evaluation.

## Methods

### Photography and facial image processing

**Figure 8:**
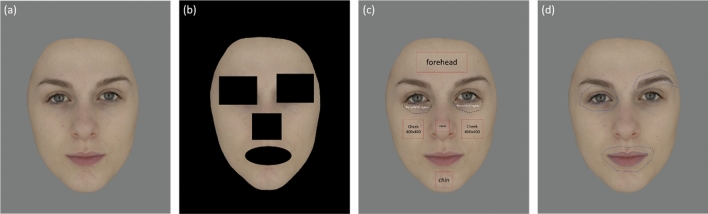
An example of the facial image and areas selected for calculating facial colour characteristics. (**a**) An example of the original facial image; (**b**) The facial area (the non-black area) used to calculate average facial colour; (**c**) Areas of interest used to calculate local skin colour and skin colour variation; (**d**) Areas of the features and the surrounding skin used to calculate facial colour contrast.

Eighty facial images, including 40 Chinese images and 40 Caucasian images with the same age range between 20 and 40 were selected from the Liverpool-Leeds Skin-colour Database (LLSD)^37^. All the facial images were captured by a digital SLR camera (Nikon D7000) in a VeriVide DigiEye® light booth, which had a mid-grey matte background and was illuminated by a D65 fluorescent simulator offering evenly diffused illumination. Each subject was asked to sit 57.5 cm in front of the camera with a neutral facial expression and their target facial area was adjusted to fit within the camera image. Images were captured and stored in uncompressed tagged image file format (.TIF) at a resolution of 3264 × 4928 pixels and 72 dots per inch (dpi). No colour correction or spatial filtering was applied to these images. After camera colour characterization, the device-independent CIE colorimetric coordinates of each pixel could be derived. For each facial image, the hair, ears, and any visible clothing were then removed, and the face was scaled to be in the centre of the image with a mid-grey background (L*, a*, b* = 50, 0, 0). An example of a Caucasian facial image is shown in Fig. 8a.

### Analysis of facial colour characteristics

In total, nineteen facial colour characteristics from three classes were analysed for each of the 80 facial images. All the areas of interest shown in Fig. 8 were selected manually for each image and all the calculations were performed in MATLAB.

#### Average facial colour and local skin colour

The average facial colour specification, in terms of CIELAB coordinates (L^*^, a^*^, b^*^), of 80 test facial images (40 Chinese and 40 Caucasian) were calculated as the overall mean of each pixel in the facial area, excluding the mouth, nose, eyes, and eyebrows, as shown in Fig. 8b. Considering the study of Jones et al.^17^, the local skin colour of cheek redness, a*, and periorbital lightness, L*, were also calculated as the overall mean of each pixel within the selected areas (Fig. 8c).

#### Skin colour variation

To access the facial skin colour variation, the mean colour difference from the mean (MCDM) was adopted, a measure commonly used to describe colour variation for a set of data points in CIELAB space, using the following equation^47,48^

$$MCDM=\frac{\sum_{i=1}^{N}{[{({L}_{i}^{*}-\overline{{L }^{*}})}^{2}+{({a}_{i}^{*}-\overline{{a }^{*}})}^{2}+{({b}_{i}^{*}-\overline{{b }^{*}})}^{2}]}^{1/2}}{N}$$

In this study, MCDM was used to evaluate skin colour variation of any target facial areas, where ${L}_{i}^{*}$, ${a}_{i}^{*}$, and ${b}_{i}^{*}$ are the CIELAB coordinates for the ith pixel of the area, $\overline{{L }^{*}}$, $\overline{{a }^{*}}$, and $\overline{{b }^{*}}$ are the average CIELAB coordinates of the facial area and $N$ is the number of pixels within the area. As outlined in Fig. 8c, the MCDM of the forehead, cheek, nose, and chin areas was calculated and the grand mean of the MCDM values of the four parts was then obtained to represent the skin colour variation over the whole facial area. Both the skin colour variation of the whole facial area (MCDM) and the cheek (MCDM-Cheek) were analysed in this study. The smaller the value of the MCDM, the smaller the colour difference and the more even/homogeneous is the skin colour distribution.

#### Facial colour contrast

Both the adapted version of the Michelson contrast and the CIELAB colour differences (∆E) were used in the present study to describe facial colour contrast between three facial features (eyes, eyebrows, and mouth) and their surrounding skin (Fig. 8d). The adapted Michelson contrast of the three dimensions (L^*^, a^*^, b^*^) was considered, as defined by the following equation,

$${C}_{\mathrm{Feature}}= \left|\frac{{A}_{\mathrm{Skin}}-{A}_{\mathrm{Feature}}}{{A}_{\mathrm{Skin}}+{A}_{\mathrm{Feature}}}\right|$$

where ${A}_{\mathrm{Skin}}$ is the respective CIELAB coordinates (L^*^, a^*^, b^*^) of the surrounding facial skin and ${A}_{\mathrm{Feature}}$ is the respective CIELAB coordinates (L^*^, a^*^, b^*^) of the facial features (eyes, eyebrows, and mouth). Meanwhile, the CIELAB colour differences (∆E) between the three facial features and their surrounding skin were also calculated and the facial colour contrast was defined by the following equation,

$$\Delta E={[{({L}_{1}^{*}-{L}_{2}^{*})}^{2}+{({a}_{1}^{*}-{a}_{2}^{*})}^{2}+{({b}_{1}^{*}-{b}_{2}^{*})}^{2}]}^{1/2}$$

where ${L}_{1}^{*}$, ${a}_{1}^{*}$, and ${b}_{1}^{*}$ are the CIELAB coordinates of the facial features, and ${L}_{2}^{*}$, ${a}_{2}^{*}$, and ${b}_{2}^{*}$ are the CIELAB coordinates of their surrounding skin area. For both ${C}_{\mathrm{Feature}}$ and $\Delta E$, the bigger the value, the larger the facial colour contrast.

### Ratings of facial preference

A psychophysical experiment was conducted to obtain the subjective ratings of facial preference regarding the skin colour of each facial image. A BenQ professional colour display, with the white point set to CIE illuminant D65, was used to reproduce the real facial images in the experiments. After display colour characterization, the CIELAB values for each pixel were transformed to display RGB values for each facial image. 44 observers, including 22 Caucasians (13 male; overall mean age ± SD = 24.27 ± 5.30 years) and 22 Chinese (7 male; overall mean age ± SD = 26.05 ± 3.96 years) evaluated the colour appearance of the 80 facial images in terms of the three attributes of facial preference: attractiveness, perceived healthiness, and visual age. The three attributes were judged in three separate sessions. Each observer was given 8 s to view each facial image and then was asked to make a judgement of the facial skin colour without a time limit. The following question was asked after the observation of each image, “Based on the skin color, what attractiveness score (or healthiness score or the estimated age, depend on different sessions) you would give for the last image?” Based on the categorical judgment method, the perceived facial attractiveness and healthiness were rated using a 7-point Likert-type scale where 1 represented ‘least attractiveness’/’healthiness’ and 7 represented ‘best attractiveness’/‘healthiness’. The visual age was rated on a single-year step scale from 1 to 99 years. The ages of subjects in the 80 images were in the range of 20–40 years although the observers were not aware of this fact.

All the observers were given instructions in English, and each gave written informed consent before the experiments took place. The Chinese observers were from mainland China, and at the time of the study, they spent 1–3 years on average in the UK as students or visiting scholars at the University of Leeds. This study was approved by the Ethics Committee at the University of Leeds (PVAR 13-057, LTDESN-090) and all methods were performed in accordance with the relevant guidelines and regulations. The informed consent was obtained for publication of identifying information/images in an online open-access publication.

### Data analysis

Separate analyses were carried out for each ethnic group to examine the colour variables that might predict each preference rating; thus, the Caucasian dataset is the preference ratings of the Caucasian images judged by the Caucasian observers and the Chinese dataset is the preference ratings of the Chinese images judged by the Chinese observers. The mean values and standard deviations of facial colour characteristics and preference ratings were first calculated for both ethnic datasets to show the range and variation of both facial colour characteristics in CA and CN facial images and the preference ratings from the two groups of observers. Inter-observer variability was then examined by calculating Cronbach Alpha Coefficients^49^. The internal consistency in the ratings of attractiveness, healthiness, and age for both the Caucasian dataset and Chinese dataset is very high, ranging from Cronbach's α = 0.90 to Cronbach's α = 0.96 for Caucasian dataset and from Cronbach's α = 0.92 to Cronbach's α = 0.96 for Chinese dataset across the attributes^16^. Ratings were averaged across all observers to create a score for each face on each preference attributes before correlation analysis and modelling from the face level colour traits. All the colour predictors were z-standardized prior to analysis. The Pearson Correlation Coefficient (two-tailed) was used to assess the relationships between the various facial colour characteristics and each of the three preference ratings: facial attractiveness, perceived healthiness, and perceived age. To further investigate the role of different classes of colour characteristics in predicting the preference of real human faces, and identify the most important colour predictors, techniques from machine learning were implemented in the modelling process. Similar approaches can be found in previous studies^38,39^.

We used cross-validation to compare the predictive power of different classes of facial colour characteristics and the analysis was done by the caret package in R^40^. During the cross-validation analysis, the data was split into a training dataset for model estimation and a testing dataset for predictive accuracy test, so that the problem of overfitting could be avoided by testing the model with the new testing data rather than the old training data. Moreover, the process was repeated many times with different random splits of the data. The model’s overall predictive fit was assessed by the mean RMSE (root mean square error) over all splits. RMSE is a statistic of predictive accuracy representing the difference between predicted values from the model and observed values from the experiments and is not inflated by the number of predictors, compared to other statistics such as R^2^.

We finally used elastic net regression^41,50^ to include all the colour variables together into one regression model and to evaluate the relative importance of different colour predictors to the preference judgements. Considering the large correlations between different colour variables, the traditional multiple regression models may cause problems of multicollinearity and result in overfitted models. The elastic net regression is a linear regression which shrink predictors to reduce overfitting through regularization and meanwhile perform variable selection by setting the coefficients of uninformative parameters to zero. The models have two hyperparameters which could be tuned to optimize the model fit, α, which controls the degree to which the model shrinks coefficients, and λ, which determines how aggressively coefficients are set to zero. We also implemented cross validation to first generate the combination of α and λ with maximized fit (minimized RMSE) and second test the model fit with the optimal α and λ by the mean RMSE over all splits.

## Supplementary Information

Supplementary Information.
