# Supplementary material for: Different colour predictions of facial preference by Caucasian and Chinese observers
Source: Sci Rep. 2022 Jul 16;12:12194. doi: 10.1038/s41598-022-15951-8 (PMC9288550; doi:10.1038/s41598-022-15951-8)
Supplement: Supplementary file 1 — Supplementary Information. [file 41598_2022_15951_MOESM1_ESM.pdf]

Appendix 1. Descriptive statistics for the facial colour characteristics and preference ratings for the Caucasian dataset (CA) and Chinese dataset (CN). t-test values for which P>0.05 are shown in bold.

	Mean (SD)		*t*-test
	CA	CN	
**Facial colour characteristics**			
L*	60.63 (2.06)	55.92 (2.10)	<0.001
a*	8.10 (1.26)	8.72 (1.16)	0.025
b*	15.25 (2.06)	18.55 (1.50)	<0.001
Cheek-a*	9.91 (1.70)	10.04 (1.91)	**0.741**
Periorbital-L*	54.77 (2.87)	53.07 (2.11)	0.003
MCDM-Cheek	2.48 (0.32)	2.40 (0.31)	**0.237**
MCDM	2.58 (0.21)	2.51 (0.22)	**0.169**
Eyes-C-L*	0.15 (0.03)	0.18 (0.03)	<0.001
Eyes-C-a*	0.21 (0.09)	0.13 (0.07)	<0.001
Eyes-C-b*	0.14 (0.07)	0.21 (0.04)	<0.001
Eyes-△E	15.02 (2.78)	18.25 (2.47)	<0.001
Brows-C-L*	0.16 (0.06)	0.13 (0.05)	0.010
Brows-C-a*	0.06 (0.05)	0.13 (0.08)	<0.001
Brows-C-b*	0.06 (0.04)	0.18 (0.04)	<0.001
Brows-△E	16.32 (5.18)	13.67 (4.17)	0.014
Mouth-C-L*	0.12 (0.02)	0.10 (0.02)	0.001
Mouth-C-a*	0.41 (0.06)	0.33 (0.06)	<0.001
Mouth-C-b*	0.14 (0.05)	0.17 (0.04)	0.002
Mouth-△E	17.84 (2.64)	15.26 (2.13)	<0.001
**Preference ratings**			
Attractiveness	3.92 (0.87)	4.04 (0.83)	
Healthiness	4.09 (0.96)	4.50 (0.82)	
Age	25.59 (2.96)	26.59 (2.51)	

Appendix 2. Zero-order Pearson correlations between facial colour characteristics and preference ratings. CN results are below the diagonal and CA results are above the diagonal. The different coloured boxes show correlation at p< 0.001, p<0.01 and p<0.05.

					average/local skin colour					skin colour variation		facial colour contrast											
		Attractiveness	Healthiness	Age	L*	a*	b*	Cheek-a*	Periorbital-L*	MCDM-Cheek	MCDM	Eyes-C-L*	Eyes-C-a*	Eyes-C-b*	Eyes-ΔE	Brows-C-L*	Brows-C-a*	Brows-C-b*	Brows-ΔE	Mouth-C-L*	Mouth-C-a*	Mouth-C-b*	Mouth-ΔE
	Attractiveness		**0.912**	**-0.343**	-0.267	-0.103	**0.338**	-0.036	-0.250	-0.300	-0.254	0.224	0.027	0.156	0.190	0.111	-0.083	0.190	0.104	**-0.321**	-0.037	**0.365**	-0.216
	Healthiness	**0.927**		-0.293	**-0.351**	-0.046	**0.381**	0.001	**-0.359**	-0.232	**-0.363**	0.104	0.030	0.098	0.049	0.070	-0.125	0.211	0.045	-0.232	-0.056	**0.356**	-0.166
	Age	**-0.828**	**-0.818**		-0.259	0.262	0.130	0.153	-0.133	**0.442**	**0.457**	0.231	-0.184	-0.003	0.192	0.098	0.184	0.102	0.070	-0.168	-0.290	0.128	-0.198
average/local skin colour	L*	**0.477**	**0.415**	**-0.571**		**-0.519**	**-0.425**	**-0.429**	**0.715**	**-0.318**	-0.296	-0.203	-0.096	**-0.371**	-0.088	-0.180	0.209	-0.253	-0.073	0.261	**0.651**	**-0.382**	**0.414**
	a*	**-0.377**	**-0.342**	**0.415**	**-0.554**		-0.301	**0.898**	-0.108	**0.329**	0.193	0.061	0.124	0.024	0.024	**-0.346**	-0.276	-0.130	**-0.411**	-0.022	**-0.616**	-0.269	-0.182
	b*	0.071	0.006	0.072	-0.387*	0.153		-0.271	**-0.570**	0.076	0.104	0.239	0.084	**0.543**	0.228	**0.428**	0.239	**0.555**	**0.411**	-0.276	-0.048	**0.735**	-0.159
	Cheek-a*	-0.247	-0.167	0.229	-0.406**	**0.881**	0.109		-0.095	**0.336**	0.181	-0.018	0.115	-0.020	-0.039	**-0.416**	-0.286	-0.139	**-0.471**	0.070	**-0.436**	-0.240	-0.040
	Periorbital-L*	0.060	0.034	-0.146	**0.538**	-0.151	**-0.458**	-0.156		-0.187	-0.189	-0.121	-0.127	**-0.401**	0.015	-0.184	0.159	-0.257	-0.119	-0.018	0.222	**-0.547**	0.066
variation	MCDM-Cheek	**-0.369**	-0.294	**0.373**	-0.191	0.134	0.167	0.221	0.052		**0.448**	0.117	-0.125	0.188	0.115	-0.031	0.256	0.196	-0.079	0.035	-0.305	0.017	-0.068
	MCDM	**-0.399**	-0.296	**0.383**	**-0.383**	0.124	0.194	0.066	-0.195	**0.510**		0.227	-0.186	0.164	0.221	0.293	0.165	0.252	0.255	-0.109	-0.240	0.137	-0.171
facial colour contrast	Eyes-C-L*	0.151	0.235	-0.095	0.143	-0.279	-0.240	-0.020	-0.088	0.111	-0.036		-0.242	**0.330**	**0.971**	**0.366**	0.309	**0.493**	**0.356**	**-0.318**	-0.147	0.058	-0.250
	Eyes-C-a*	-0.089	-0.069	0.018	-0.266	**0.324**	0.270	0.303	-0.076	0.246	0.196	-0.196		**0.465**	-0.159	-0.117	-0.198	-0.225	-0.117	0.241	-0.033	-0.141	0.190
	Eyes-C-b*	0.310	**0.350**	**-0.409**	0.119	-0.164	0.174	0.017	-0.267	0.055	-0.150	0.239	**0.470**		**0.393**	0.262	0.089	**0.315**	0.235	-0.177	-0.195	0.237	-0.153
	Eyes-ΔE	0.273	**0.338**	-0.254	**0.337**	**-0.383**	-0.269	-0.093	0.059	0.079	-0.120	**0.963**	-0.151	**0.356**		**0.360**	**0.376**	**0.481**	**0.365**	**-0.332**	-0.107	0.004	-0.236
	Brows-C-L*	-0.265	-0.215	**0.351**	-0.181	-0.106	-0.195	-0.097	0.176	0.234	0.102	**0.314**	-0.265	**-0.334**	0.206		0.253	**0.452**	**0.990**	-0.300	0.079	**0.367**	-0.215
	Brows-C-a*	**-0.472**	**-0.406**	**0.575**	**-0.380**	-0.021	-0.292	-0.050	-0.042	0.169	0.180	0.248	-0.202	**-0.428**	0.083	**0.761**		0.206	0.278	-0.125	0.163	0.075	0.024
	Brows-C-b*	-0.238	-0.159	0.277	0.012	-0.262	**-0.421**	-0.196	**0.361**	0.184	0.110	0.295	-0.201	**-0.328**	0.234	**0.681**	**0.798**		**0.420**	-0.265	-0.063	0.230	-0.173
	Brows-ΔE	-0.211	-0.168	0.307	-0.085	-0.181	-0.220	-0.159	0.262	0.239	0.078	0.310	-0.297	**-0.354**	0.223	**0.989**	**0.750**	**0.736**		-0.267	0.159	**0.356**	-0.158
	Mouth-C-L*	0.057	0.122	-0.090	0.239	-0.250	-0.242	-0.162	0.106	0.043	0.036	**0.369**	-0.278	0.002	**0.360**	0.091	0.058	0.209	0.118		**0.495**	**-0.435**	**0.915**
	Mouth-C-a*	**0.526**	**0.511**	**-0.535**	**0.517**	**-0.635**	-0.198	**-0.522**	0.074	**-0.514**	**-0.360**	0.215	**-0.500**	0.086	0.303	0.014	-0.079	0.125	0.065	**0.316**		-0.170	**0.750**
	Mouth-C-b*	0.229	0.171	-0.054	-0.125	-0.174	**0.496**	-0.189	**-0.416**	-0.149	0.137	-0.018	-0.180	0.077	-0.029	-0.028	-0.066	-0.197	-0.032	-0.020	**0.372**		**-0.358**
	Mouth-ΔE	**0.438**	**0.444**	**-0.424**	**0.468**	**-0.452**	-0.082	**-0.343**	0.050	**-0.370**	-0.172	0.234	**-0.492**	0.023	0.296	-0.026	-0.134	0.073	0.027	**0.624**	**0.859**	**0.470**	

p<0.001
p<0.01
p<0.05
